# Effect of Tonic Pain on Motor Acquisition and Retention while Learning to Reach in a Force Field

**DOI:** 10.1371/journal.pone.0099159

**Published:** 2014-06-09

**Authors:** Mélanie Lamothe, Jean-Sébastien Roy, Jason Bouffard, Martin Gagné, Laurent J. Bouyer, Catherine Mercier

**Affiliations:** 1 Centre interdisciplinaire de recherche en réadaptation et intégration sociale (CIRRIS), Quebec City, Quebec, Canada; 2 Département de réadaptation, Faculté de médecine, Université Laval, Quebec City, Quebec, Canada; University of Bologna, Italy

## Abstract

Most patients receiving intensive rehabilitation to improve their upper limb function experience pain. Despite this, the impact of pain on the ability to learn a specific motor task is still unknown. The aim of this study was to determine whether the presence of experimental tonic pain interferes with the acquisition and retention stages of motor learning associated with training in a reaching task. Twenty-nine healthy subjects were randomized to either a Control or Pain Group (receiving topical capsaicin cream on the upper arm during training on Day 1). On two consecutive days, subjects made ballistic movements towards two targets (NEAR/FAR) using a robotized exoskeleton. On Day 1, the task was performed without (baseline) and with a force field (adaptation). The adaptation task was repeated on Day 2. Task performance was assessed using index distance from the target at the end of the reaching movement. Motor planning was assessed using initial angle of deviation of index trajectory from a straight line to the target. Results show that tonic pain did not affect baseline reaching. Both groups improved task performance across time (p<0.001), but the Pain group showed a larger final error (under-compensation) than the Control group for the FAR target (p = 0.030) during both acquisition and retention. Moreover, a Group x Time interaction (p = 0.028) was observed on initial angle of deviation, suggesting that subjects with Pain made larger adjustments in the feedforward component of the movement over time. Interestingly, behaviour of the Pain group was very stable from the end of Day 1 (with pain) to the beginning of Day 2 (pain-free), indicating that the differences observed could not solely be explained by the impact of pain on immediate performance. This suggests that if people learn to move differently in the presence of pain, they might maintain this altered strategy over time.

## Introduction

Pain is one of the most common and disabling symptoms following injury to the peripheral (e.g. amputation) or central nervous system (e.g. spinal cord injury or stroke). In motor rehabilitation programs, a large proportion of patients receiving intensive training to improve their upper limb function experience neuropathic pain. Epidemiologic data indicate that compared to patients with similar injuries but without associated pain, patients experiencing pain exhibit poorer motor outcomes, suggesting that they might have limited ability to relearn previous motor patterns, or to learn new ones in order to compensate for residual deficits. For example, four months after a stroke, 32% of patients suffer from moderate to severe pain,[1] and pain is associated to a poorer sensorimotor recovery.[2], [3] After an amputation, pain affects 50–80% of patients,[4], [5] and is associated with less success in learning to use a prosthesis efficiently.[6], [7]

In parallel, studies using experimental pain in humans and animals have shown that pain exerts modulatory influences over the activity in motor pathways. It has been demonstrated that pain can lead to a reduction of maximal voluntary contraction, a decrease in endurance and changes in coordination during dynamic motor tasks (see [8]–[11] for reviews), although the cause of these alterations in motor function are still not fully understood. Several studies have also investigated the influence of pain on corticospinal excitability by looking at motor responses evoked by transcranial magnetic stimulation (TMS) applied over M1.[11]–[13] Most of these studies reported that different types of acute experimental pain applied within or close to the target muscle exert an inhibitory influence on corticospinal excitability.[14]–[26] Some studies have obtained apparently contrasting results, reporting motor facilitation [27]–[29]. However, in the latter, motor evoked potentials were either recorded in active muscles (while matching the force level, and not the level of EMG) or in muscles proximal to the application of pain, which might explain the differences observed. The corticospinal inhibition generally observed in the presence of pain might hamper optimal neural activation and immediate motor performance. But even more importantly, it might impair the plastic potential of the motor cortex and the ability to learn, or relearn in the case of rehabilitation, a motor task. Evidence supporting this view comes from a study in which healthy volunteers were trained in a tongue-protrusion task, with or without local application of capsaicin.[30] Although participants improved their performance in the motor task following training in both the painful and nonpainful conditions, the improvement was significantly lower in the presence of pain. In addition, pain suppressed the training-induced motor plasticity effects (e.g. increased excitability) observed in the control condition. It is not possible however to determine whether the lack of corticospinal facilitation was caused by poorer motor performance, or was a result of a lower/less effective training in response to pain (the number of trials was similar between groups, but not necessarily the intensity/duration of each trial). Moreover, contrasting results have been obtained by Ingham et al.,[31] who did not observed any effect of local pain (resulting from hypertonic saline injection into FDI) on the plasticity induced by repetitive finger movements.

An important question regarding the effect of pain on motor learning concerns its impact on the retention of performance. To establish that a novel task has been learned, other criteria need to be met beyond a simple improvement in performance occurring within a training session. Indeed, motor learning involves several steps: improvement in performance during training (acquisition), transfer to longer-term memory (consolidation), and the ability to recall the stored motor memory (retention).[32] In the studies presented above, motor improvement was observed in the presence of pain, but motor retention was not assessed. It is well known from the motor learning literature that motor gains observed during training are not always predictive of longer-term gains.[33] From a clinical perspective, it is the retention of the learned behaviour that is the critical outcome, much more than the improvement during the therapy itself.

Application of low-frequency (inhibitory) repetitive TMS to M1 prior to force field exposure has been shown to disrupt the retention of this type of learning when tested 24 h later, without affecting performance during the acquisition phase. It suggests that M1 might be important for initiating the development of long-term motor memories.[34] It has been hypothesized that motor cortex inhibition induced by pain might reflect a ‘partial motor decerebration’ in order to promote spinal protective reflexes.[12] Pain might therefore potentially exert an effect on M1 that is similar to the inhibition induced by low-frequency repetitive TMS. If M1 plays an important role in the development and consolidation of motor memories, it can then be hypothesized that pain might affect these processes and thereby interfere not only with acquisition during motor learning, but also (and perhaps more importantly) with consolidation and retention of motor memories.

The general objective of this study was to determine whether the presence of experimental tonic pain interferes with motor learning associated with training in a reaching task. To achieve this goal, we compared two experimental groups (Control and Pain) during a force field adaptation task performed on two consecutive days. Subjects were tested during both baseline and perturbed reaching movements on Day 1 (with or without pain), and only in the perturbed movement condition without pain on Day 2. Specific objectives were to determine the effect of tonic pain on motor performance during: [1] an unperturbed reaching task (baseline performance); [2] the acquisition stage of learning (i.e. change in performance between the beginning and the end of force-field exposure on Day 1); [3] the retention stage of learning (i.e. movement kinematics at the beginning of force-field exposure on Day 2 compared to Day 1). A tonic pain model (induced experimentally using capsaicin cream) that does not occur/increase in relation to specific movements was selected as this type of pain is frequent in patients with neuropathic pain who face major motor learning challenges during rehabilitation, such as patients with spinal cord injury, amputation or stroke. The long-term goal is to understand how pain affects motor learning abilities in these clinical populations. However, studying experimental acute pain in healthy subjects is a necessary first step to control for potential confounds that would be faced in populations with neuropathic pain, such as the presence of lesions to the central nervous system, alterations in sensory feedback, peripheral biomechanical constraints, sleep disorders, medication, etc.

## Methods

### Ethics statement

**Figure 1:**
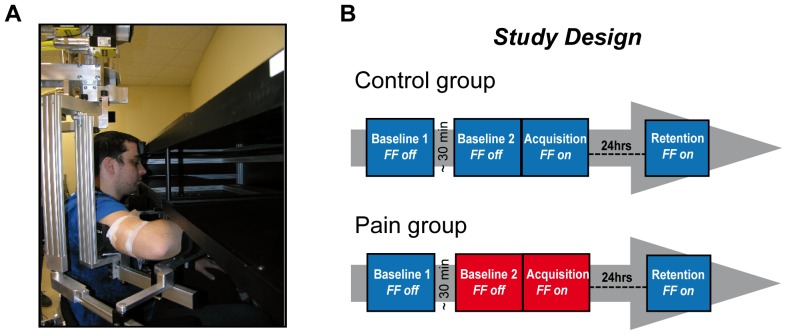
Experimental set-up and design. **Panel A** - Subjects made right arm ballistic reaching movements to visual targets using the KINARM robotic exoskeleton. The zone on the upper arm where the capsaicin was applied is shown. **Panel B** - On Day 1, two blocks of baseline measurements (without force field) were recorded for both groups. The Acquisition period (with force field perturbation) began immediately after the end of Baseline 2. On Day 2, only one block with the force field was performed to evaluate the Retention. Each block includes 50 trials/target. For the Pain group, the red indicates when the capsaicin was present (single application between Baseline 1 and 2).

The ethical review board of the Institut de réadaptation en déficience physique de Québec (IRDPQ, Québec, Canada) approved the study. All participants provided their written informed consent prior to inclusion. The individual appearing on Figure 1 in this manuscript has given written informed consent (as outlined in PLOS consent form) to publish his photograph.

### Sample and study design

Thirty right-handed healthy individuals with no reported history of neurological or musculoskeletal problems that could affect performance during the task were recruited and randomized to either the Control group or the Pain group. One subject from the Control group was excluded from the analyses because he reported shoulder pain at the end of the experiment on Day 1.

Experiments were carried out on two consecutive days. The reaching task was assessed using a KINARM robotized exoskeleton (BKIN Technologies, Canada), that allows force field application at a given joint.[35] On Day 1, subjects performed the reaching task before and during force field exposure. Two blocks of baseline measurements (without force field) were recorded for both groups. Experimental pain (for the Pain group) was induced between the two baseline measurements. Together, the two baseline measurements therefore allowed assessing whether pain altered the initial motor pattern (*Objective 1*). Immediately after the last reaching trial in the second baseline assessment, the force field was unexpectedly applied (subjects were aware that a perturbation was going to be applied, but the moment and the nature of the perturbation were unknown) and the subjects had to adapt to the resulting perturbation (*Objective 2*, Acquisition). Note that during Acquisition, subjects in the Pain group were still experiencing pain. On Day 2, subjects performed the reaching task only during exposure to the force field (*Objective 3*, Retention – the same force field as Day 1, and subjects were not informed prior to the first reaching movement that the force field was turned on). No wash-out period was performed at the end of Day 1, and no baseline measurement without the force field was performed on Day 2, as it can reduce retention.[36] No pain was applied on Day 2. Figure 1 summarizes the study design.

### Experimental pain

Subjects were informed of their group assignment after Baseline 1. The experimental tonic pain was induced with a single topical application of 1% capsaicin cream. A thin layer (∼1 mm) of cream forming a 1 cm-wide ring was applied around the upper arm (just above the elbow) of the trained limb. Once the cream was applied, subjects had to verbally rate their pain intensity every 3 minutes using a numeric rating scale (NRS) ranging from 0 (no pain) to 10 (the worst pain that can be imagined). The experiment resumed when pain reached a plateau (average of 25.1±3.9 minutes). A wait period of 30 minutes was also imposed to the Control group between Baseline 1 and Baseline 2.

### Task description and instrumentation

Subjects made right arm ballistic reaching movements to visual targets using the KINARM robotic exoskeleton. They performed the reaching movements, with the arm in the horizontal plane, to one of two targets (random sequence) located 10 cm away from the central starting position (one at 120° (FAR) and one at 300° (NEAR) – i.e. requiring multijoint coordination of the elbow [flexion-extension] and shoulder [horizontal adduction-abduction]). The central starting position was determined in order to achieve a standardized posture of 50° of horizontal abduction [with respect to the sagittal plane] and 90° of elbow flexion). Note that the KINARM exoskeleton allows movement at the elbow and the shoulder, but not at the wrist. Visual feedback of index position, start position, and targets was presented in the same plane as the arm using an overhead projector and a half-silver mirror.

From the starting position, subjects were instructed to “shoot” through the target as quickly and precisely as possible, beginning their movement as soon as the target appeared in the virtual environment. As soon as the index crossed an invisible 10-cm radius circle centered on the starting position, the robot produced a dampening field to rapidly stop the movement.[37], [38] The robot then actively returned the hand to the start position. The color of the target at the end of the trial provided feedback to the subject on both the precision and the time needed to cross the 10 cm radius. This visual feedback was important especially with regard to the speed of the movement, in order to obtain ballistic movements with similar velocities (and hence similar force field magnitudes) across conditions and subjects. For a trial to be considered successful, the movement duration had to be below 700 ms (including reaction time). If the duration of the movement was below 700 ms and the index hit the target, the target turned green. If the duration of the movement was below 700 ms but the index missed the target, it turned yellow. Finally, if the duration of movement was too long, the target turned red.

One hundred trials (50/target) were performed in each period (i.e. for each Baseline 1 and 2, Acquisition and Retention). A velocity-dependent resistive force (−3 Nm·s/rad) was applied at the elbow by the KINARM during the Acquisition and Retention periods. Joint angular positions of the elbow and shoulder were obtained from KINARM motor encoders and sampled at 1 kHz. The position of the index was computed in real-time by the Dexterit-E software of the KINARM system. Data processing was made with Matlab (MathWorks, R2011b).

### Variables and analyses

**Figure 2:**
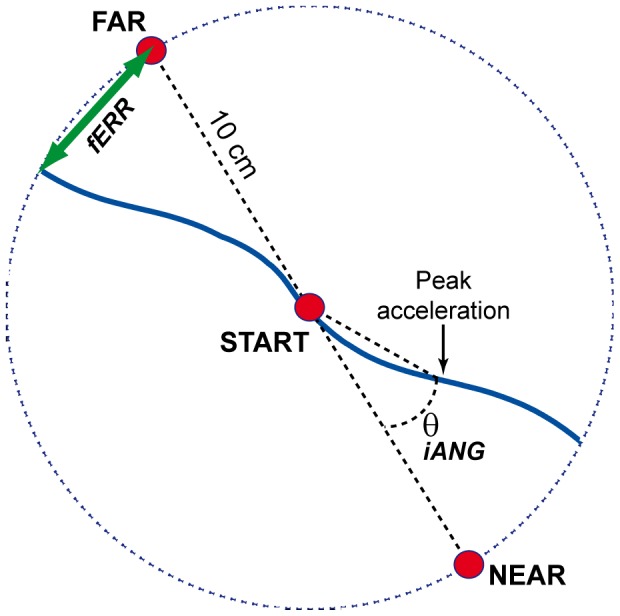
Selected kinematic variables. This figure depicts the kinematic variables extracted from index finger trajectories, using examples of typical trials early in the Acquisition period (i.e. trajectories are strongly deviated in the direction of the force field). The fERR is measured as the distance between the index and the target when the index crossed the invisible 10-cm radius circle centered on the starting position. The iANG is computed as the angle between: 1) a line joining the position of the index at movement onset and the target; and 2) another line joining the position of the index at movement onset and at its first peak of acceleration.

Two main variables were used to assess motor performance: the initial angle of deviation (iANG) and the final error (fERR). The iANG reflects subject's motor planning as it is based on the initial part of the movement only (prior to the first peak of acceleration). iANG was computed as the angle between: 1) a line joining the position of the index at movement onset and the target; and 2) another line joining the position of the index at movement onset and at its first peak of acceleration (see Figure 2). As the first peak of acceleration occurs early in the index trajectory, the initial angle of deviation is not likely to be influenced by sensory feedback. The fERR was used as an indicator of task performance, as the explicit aim of the task was to “shoot” through the target with the index as precisely as possible. It was measured as the distance between the index and the target when the index crossed the invisible 10-cm radius circle centered on the starting position. It is important to note that both variables are signed values, negative values reflecting an under-compensation for the force-field (i.e. a deviation in the direction in which the limb is pushed by the force-field), while positive values reflect an over-compensation.

As the force field applied was velocity-dependent, average movement speed (total index distance/total movement time (excluding reaction time)) was also computed and analyzed to ensure a comparable force-field perturbation during the Adaptation phase on both days.

Each variable was plotted as a function of trial number to obtain time course curves. The following sections of the time course curves were then defined and used for statistical comparisons:

- Baseline 1 and 2 – last 10 trials of each Baseline period.
- Early Adaptation - trials 2 to 11 of the Adaptation period (e.g. during force-field application; Day 1 or 2) (the first trial was never included in the analysis window as the force field was turned on unexpectedly).
- Late Adaptation - last 10 trials of the Adaptation period (e.g. during force-field application; analyzed for Day 1 only).

Descriptive statistics are reported as mean ± standard deviation. Statistical analyses for all variables consisted of three-way repeated measures ANOVAs to evaluate the between-group effect (Pain vs. Control), and the within group effects of Target (NEAR/FAR), and Time. For Objective 1, Time factor had two levels, Baseline 1 and Baseline 2. For Objectives 2 and 3 (tested in the same analysis), Time factor had three levels, Early Adaptation Day 1, Late Adaptation Day 1 and Early Adaptation Day 2. Post-hoc analyses were performed using a Sidak correction for multiple comparisons. All statistical analyses were performed using SPSS 13 software (SPSS Inc., Chicago, IL, USA).

## Results

The Control (n = 14, 8 females, aged 26.6±4.8) and Pain Groups (n = 15, 7 females, aged 25.8±4.1) were similar in terms of age or sex. During the application of pain on Day 1, the average pain intensity was of 7.8±0.9 at the beginning of Baseline 2 and of 7.5±1.0 at the end of the Adaptation period. Pain was therefore at a high level and remained stable throughout the experiment.

### Effect of pain on baseline motor performance

ANOVAs performed to assess the impact of pain on baseline performance revealed no effect of Group or Time x Group interaction on either iANG or fERR, which shows that pain induction between both baselines for the Pain group did not alter motor performance. The only significant difference was found between targets for both iANG (p = 0.003) and fERR (p<0.001), reflecting small differences in the movement kinematics.

The fact that pain had no impact on baseline motor performance is essential to the interpretation of subsequent analyses, especially considering that the Pain group was tested with pain on Day 1 (during acquisition), and without pain on Day 2.

### Effect of pain on motor acquisition and retention

**Figure 3:**
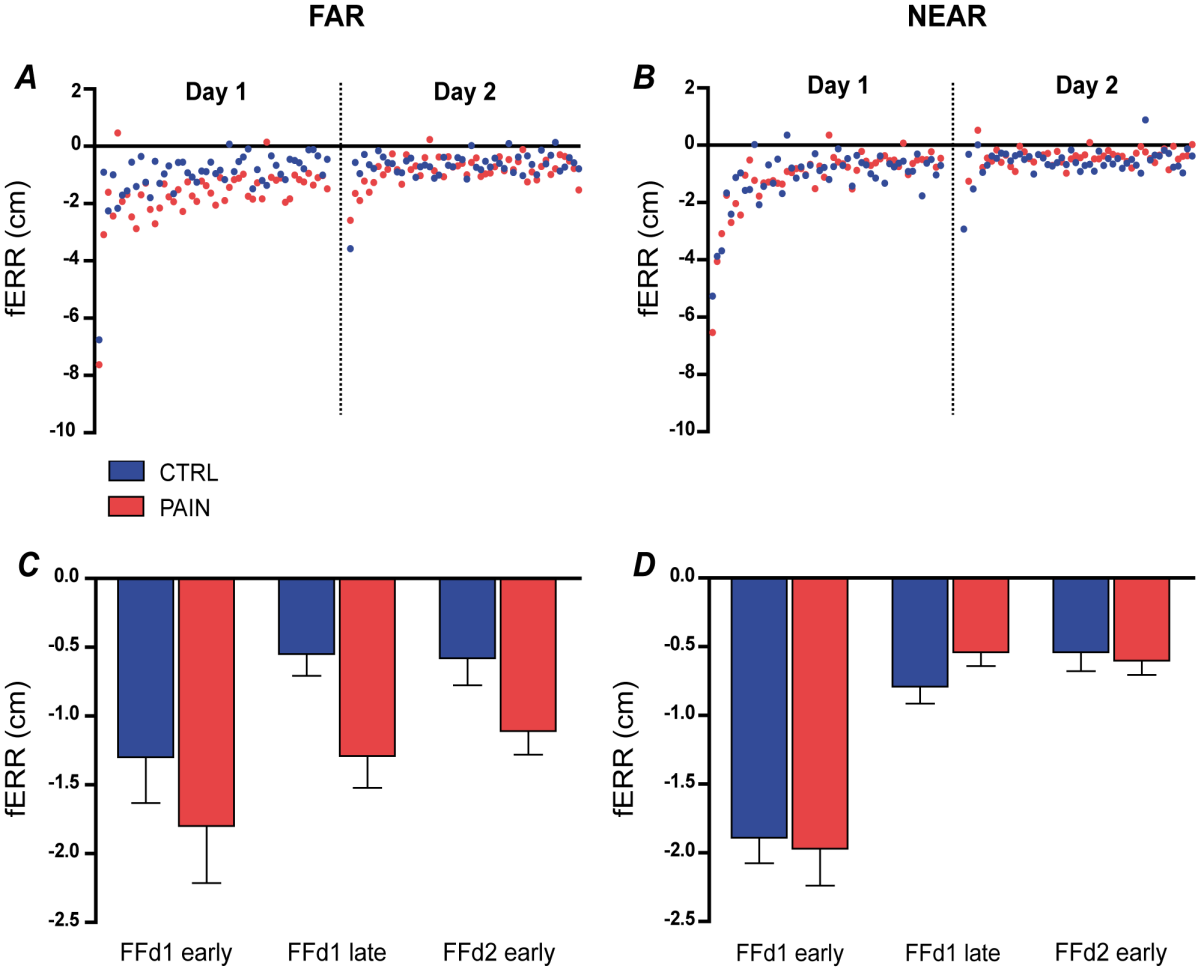
Changes in mean error (fERR) across time for each group. **Panel A** shows the time course for the FAR target, and **Panel B** for the NEAR target. **Panels C and D** show the average fERR for each group on sections of the time course selected for statistical analyses (Early/Late Day 1 and Late Day 2), respectively for FAR and NEAR target. Both groups improved their performance on Day 1, as illustrated by the rapid decay of fERR over trials. A strong Retention is demonstrated by a better performance on Early Day 2 compared to Early Day 1. However while both groups show very similar performance for the NEAR target, the Pain group systematically shows larger errors for the FAR target. This difference is still observed on Day 2, when all subjects are tested pain-free. Error bars show the standard error of mean (SEM).

**Figure 4:**
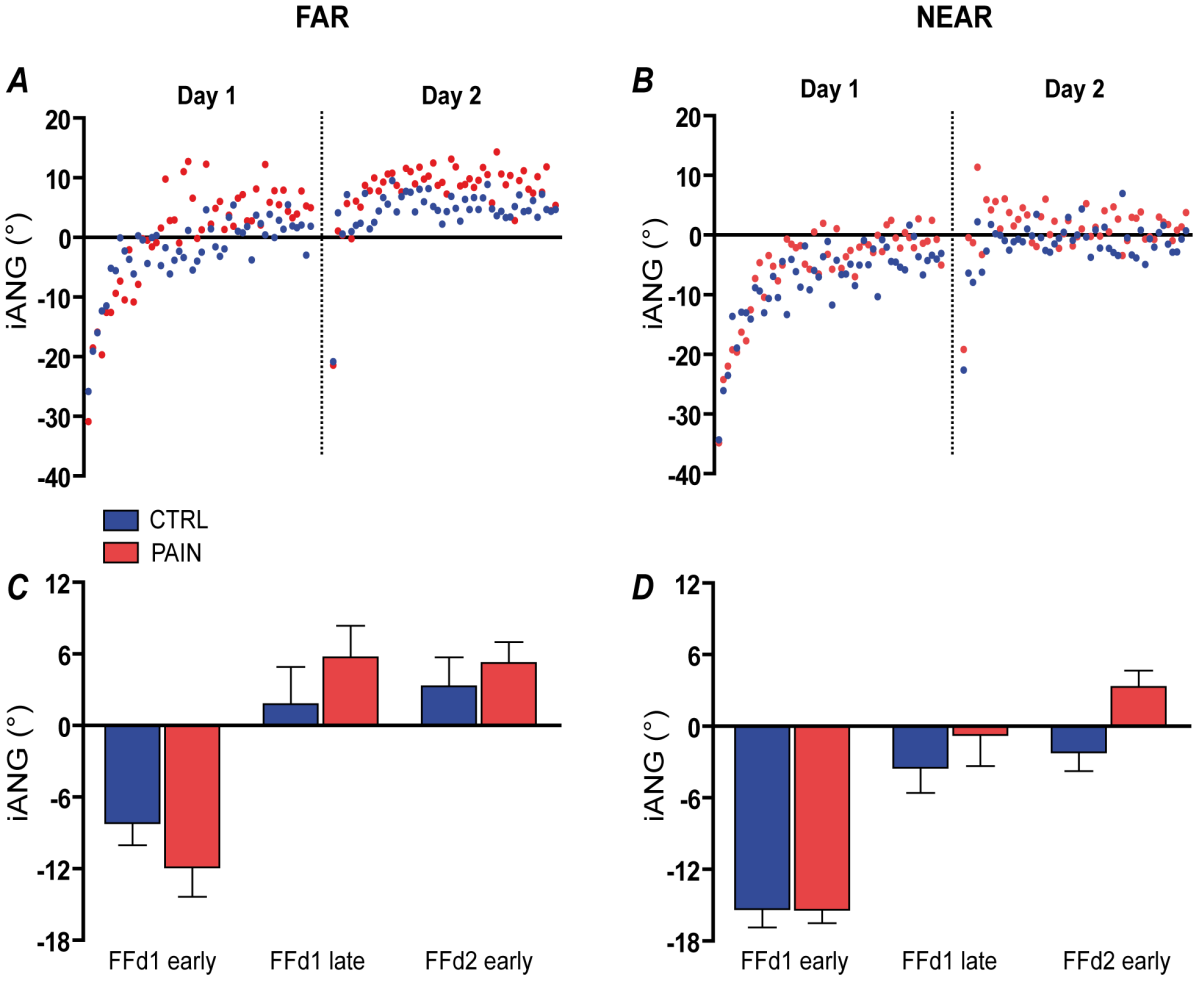
Changes in initial angle of deviation (iANG) across time for each group. **Panel A** shows the time course for the FAR target, and **Panel B** for the NEAR target. **Panels C and D** shows the average iANG for each group on sections of the time course selected for statistical analyses (Early/Late Day 1 and Late Day 2), respectively for FAR and NEAR target. On Day 1, both groups exhibited a reversal of the direction of their iANG: early in the force field their hand was deflected in the direction of the force field (negative angle value). Progressively, the movement was initiated in the opposite direction in anticipation of the perturbation. At the beginning of Day 2 iANG direction was opposite to the deviation produced by the force field and similar to what was seen at the end of Day 1, demonstrating the retention of the feedforward strategy acquired on Day 1. Error bars show the standard error of mean (SEM).

On Day 1 (acquisition), the force field perturbation induced substantial movement errors for both groups in the early period of Adaptation. Rapidly, task performance improved and motor strategy adjusted as outlined by the time course of these variables (Figures 3 and 4). On Day 2 (retention), both variables returned to the point at which they were at the end of Day 1 as early as on the second reaching movement in the force field (Figures 3 and 4, panels A and B).

For iANG, there was no Group effect (p = 0.249), while the expected main effect of Time was observed (p<0.001). Post-hoc analysis for the main effect of Time showed that subjects exhibited a reversal of the direction of their initial deviation during the Acquisition phase (Day 1 Late adaptation vs. Day 1 Early adaptation; p<0.001): early in the force field the hand was deflected in the direction of the force field, but later they started to initiate their movement in the opposite direction in anticipation of the perturbation (Figure 4). Next-day retention of this strategy was demonstrated by a significant difference between Day 1 Early adaptation and Day 2 Early adaptation (p<0.001), and absence of difference between Day 1 Late adaptation and Day 2 Early adaptation (p = 0.246). This phenomenon was more pronounced in the Pain group as outlined by a Time X Group interaction (p = 0.028). The post-hoc comparisons showed no statistically significant difference between groups at any time point, although a trend was observed on Day 2 (p = 0.074; Day 1 Early p = 0.345, Day 1 Late p = 0.227). Nevertheless the presence of a significant interaction showed that the strategy of both groups evolved differently over time, subjects from the Pain group making larger feedforward adjustments in anticipation of the force field perturbation.

For fERR, analyses showed no Group effect (p = 0.100) and the expected significant main effect of Time (p<0.001). Further a Target x Group interaction (p = 0.035) was observed (Figure 3). Post-hoc analysis for the main effect of Time showed that, for both groups, task performance improved during the Acquisition phase (Day 1 Late adaptation vs. Day 1 Early adaptation; p<0.001). Next-day retention was demonstrated by a significant difference between Day 1 Early adaptation and Day 2 Early adaptation (p<0.001), and absence of difference between Day 1 Late adaptation and Day 2 Early adaptation (p = 0.648). This reflects that both groups decreased their final error during Acquisition, and exhibited retention on Day 2. However the decomposition of the Target x Group interaction revealed a systematic difference between groups for the FAR target (p = 0.030; no difference was observed for the NEAR target, p = 0.812). The Pain group showed more negative (counter-clockwise) final error for the FAR target, indicating that they systematically under-compensated for the effect of the force field.

The analysis of average movement speed showed only a significant effect of Time (p<0.001). Post-hoc analysis indicated that movement speed was significantly smaller (p<0.01) on Day 1 Early adaptation (37.0 cm/s±1.8), but did not differ (p = 0.255) between Day 1 Late adaptation (40.3 cm/s±1.9) and Day 2 Early Adaptation (41.5 cm/s±2.0). The lack of difference between groups ensures that no systematic difference in the velocity-dependent force-field exposure can account for the differences observed between groups on the other variables.

## Discussion

Results of this study show that the presence of a significant level of acute tonic pain (∼7.5/10) did not influence baseline motor performance in a simple reaching task. Moreover subjects still significantly improved their performance in a new reaching adaptation task when training with pain, and exhibited retention when tested pain free 24 hours later. However two main differences were observed between the Pain and Control Groups. First, the Pain group showed larger changes in their feedforward strategy to minimize movement error in the force field over time, irrespective of the target. Second, the Pain group exhibited a systematic under-compensation for the force field in their final errors for one of the two targets (FAR).

### Effect of pain on baseline reaching motor performance

The large majority of previous studies that assessed the impact of pain on upper limb motor control have been designed to understand the impact of musculoskeletal pain. In these studies, pain was applied to a very focused region, often modulated by the movement itself, and induced no or only slight kinematics changes.[39]–[44] The capsaicin pain model used in the present study is more consistent with neuropathic pain, a type of pain that is unrelated to movement as well as more constant and diffuse. To our knowledge only one previous study used capsaicin, and reported no impact on upper limb kinematics [45], which is consistent with our results.

### Effect of pain on motor strategy during acquisition and retention

Interestingly, the evolution of the motor strategy over time to attain the task's goal differed between groups. When they initially faced the force field on Day 1, the Pain group tended to be more deviated in the direction of the force field than the Control group, despite experiencing similar force field intensity. Progressively both groups modified their motor planning to counteract the force field, and initiated their movement trajectory at an angle (away from a straight line) opposite to that of the force field deviation. This change in iANG was larger for the Pain group, resulting in a significant Time x Group interaction. This result indicates that subjects in pain increased more their feedforward preparation for the perturbation. Why would subjects with pain adopt a different strategy to achieve the same global result? As the same phenomenon was observed for both the FAR and NEAR targets, it seems unlikely that this pattern resulted from a different impact of pain on flexor vs. extensor muscles. One potential explanation is that subjects in pain use a larger adjustment in the feedforward component of movement to avoid the need for subsequent online error correction. Online corrections require proprioceptive information, and several studies suggest that experimental pain affects proprioception.[46]–[48]

A few studies have investigated the role of proprioceptive feedback in adaptation to force fields during reaching, and have shown that visual feedback can largely compensate for deficient proprioceptive feedback.[49]–[52] A very interesting observation comes from a study in which the effect of visual feedback (visual feedback about whole movement trajectory vs. feedback about final error only) on force field adaptation was assessed.[49] They found that although both conditions led to comparable terminal accuracy, this accuracy was achieved differently across conditions: with visual feedback, adapted trajectories in force fields were straight whereas without it, they remained curved (which was evidenced partly by larger initial angles). These results suggest that trajectory shape is influenced by the calibration of available sensory feedback signals. As pain was found to influence the evolution of trajectories across trials in the present study, either directly or via an impact on proprioceptive feedback, it would be of interest to assess the effect of pain on motor learning in a context where the subjects cannot rely on visual feedback.

### Effect of pain on task performance during acquisition and retention

In this experiment, fERR, used to assess subjects' task performance, showed an impact of pain both during the acquisition (with pain) and on next-day retention (tested pain-free). The fact that the differences observed remained on Day 2 indicates that increased errors cannot solely be explained by the impact of pain on immediate performance. It rather suggests that pain interfered with the acquisition process itself. Importantly however, this deficit was observed only for the FAR target. How can differences between targets be explained? Adapting to the force field for the FAR target does not appear to be a more challenging task, as the errors observed for the Control group on Early Day 1 were not statistically different than for the NEAR target. However both targets required different patterns of multijoint coordination. Reaching towards the NEAR target involved horizontal abduction (average excursion of 21.2±1.3) combined with elbow flexion (20.4±1.1), while the FAR target required horizontal adduction (20.9°±1.2) combined with elbow extension (25.4°±1.7). The fact that the FAR target required more elbow excursion, and that both pain and the force field were acting at the elbow, might contribute to explain the observed results. A recent study has shown that the patterns of motor control adaptation to a noxious stimulation (hypertonic saline injection) differ according to the task performed.[53] In this study, no changes were observed in motor tasks involving fewer degrees of freedom, which might be explained by the limited potential to use an alternative strategy or by the high cost associated with such compensation. These results cannot be directly translated to the context of our study, as both the pain model and the experimental paradigm employed (lower limb tasks, without a learning component) were very different. Nevertheless, they illustrate the fact that the task constraints can interact with the strategy used in the presence of acute pain.

Such task dependency of motor control adaptation to pain might also contribute to explain discrepancies between the results obtained in previous studies. Boudreau et al. showed that local pain induced by capsaicin interfered with motor acquisition during a tongue protrusion tracking task.[30] In this study,[30] capsaicin was applied directly on the effector (tongue) that applied force on a lever. During the execution of this type of tasks, increased level of pain during practice have been reported.[54] As a result, the difference in motor behaviour might reflect a strategy to minimize pain (such as undershooting or decreasing the duration of the effort). Using a different pain model (hypersaline injection in agonist muscle), Ingham et al. [31] observed no impact of pain on the rate of improvement during training on a task requiring brisk finger movements when proper feedback was provided to ensure comparable levels of training in the presence vs. absence of pain. However, as this study focused mainly on plastic changes within M1 rather than on behavioural improvement, the motor task selected was much simpler than in the present study or that of Boudreau et al. The differences highlighted between the few existing studies emphasize the need for more studies investigating different pain models (including pain related or not to the performed movement) and different types of motor tasks, as the impact of pain on learning appears likely to vary according to these factors.

### Maintenance of performance/strategy in the pain-free state

Interestingly, behaviour of the Pain group was very similar from the end of Day 1 (with pain) to the beginning of Day 2 (pain-free). This indicates that the differences observed between groups cannot be explained solely by the impact of pain on immediate performance. This is a clinically relevant observation as it suggests that motor strategies developed in the presence of acute pain might be maintained over time. It has been argued that departure from “normal” movement patterns in response to pain may not be ideal and might lead to detrimental effects in the long term, although the alternative strategy employed is effective in the short term to achieve the task's goal.[10] Although this remains speculative, long-term detrimental effects could be due to factors such as increased or modified load, decreased movement amplitude, decreased movement variability, etc.[10] Interestingly, our results show that even tonic widespread pain, which cannot be avoided by altering the movement pattern, can result in persistent alterations in motor performance.

## Conclusions

In conclusion, the results of this study show that tonic pain: 1) had no impact on baseline reaching performance; 2) resulted in more final error (under-compensation) for one target in both the acquisition and retention phases of learning during reaching tasks perturbed by a force-field; 3) resulted in a slightly altered motor strategy consisting in a larger adjustment in the feedforward component of the movement. Importantly, the strategy and performance of Day 1 carried over to the next day, despite the fact that subjects were retested in the absence of pain. This is an important observation as it suggests that even in the case of short-duration pain, moving differently while in pain can have an impact on how people will move subsequently even when pain is gone.

Comparison of these results with previous studies stresses the need for more studies on the effect of pain on motor learning to investigate the effect of different pain models, but also of different motor tasks involving visual feedback or not.
